# Comparative analysis of psychological well-being and emotional education in graduate students

**DOI:** 10.12688/f1000research.141849.1

**Published:** 2023-10-24

**Authors:** Jenniffer Sobeida Moreira-Choez, Tibisay Milene Lamus de Rodríguez, Eduardo Javier Espinoza-Solís, Graciela Josefina Castro-Castillo

**Affiliations:** 1 Posgrado, State University of Milagro, Milagro, Guayas, 091706, Ecuador

**Keywords:** Emotional intelligence, psychological well-being, emotional education, educational strategies, graduate students

## Abstract

**Background:** The growing importance of emotional intelligence in academic and professional contexts has generated a need to explore its linkage with psychological well-being. Furthermore, understanding how various demographic and academic factors can influence students' emotional perception and management is crucial for optimizing educational and intervention strategies. In this context, the primary purpose of this study was to analyze the existing relationship between emotional education and psychological well-being in graduate students.

**Methods:** The objective was to conduct a comparative analysis of perceived emotional intelligence (PEI) in different study programs offered at a specific university. The methodology, framed within a positivist paradigm, was based on a quantitative approach and examines the responses of 1,522 university students using the Trait Meta-Mood Scale (TMMS-24).

**Results:** This tool, which is divided into three dimensions (emotional attention, emotional clarity, and emotional repair), was analyzed using descriptive statistics, correlation analysis, and ANOVA tests to determine demographic and academic influences on the scores. The findings indicate deficiencies in the areas of Emotional Attention and Emotional Repair, contrasting with a marked prevalence in Emotional Clarity. Variables such as sex, age, and field of study demonstrated an influence on the dimensions of PEI. Notably, significant differences in emotional perception were found between sex and academic fields.

**Conclusions:** Specifically, training directed towards empathy proved to be a prominent factor in the perception of emotional competencies. This study highlights the influence of demographic and academic variables on emotional competencies, underscoring the need to adapt strategies in education and therapy.

## Introduction

In recent years, emotional education has garnered considerable attention across various societal sectors. Zhang and Adegbola (2022) define emotional education as the capability to identify, comprehend, and manage one’s and others’ emotions, a fundamental skill for the holistic development of individuals. It fosters healthy interpersonal relationships and effective handling of intricate emotional situations.

Despite the escalating focus on emotional education, noteworthy gaps exist in contemporary research, particularly regarding its implication for graduate students. Recognizing its pivotal role in their academic journey, there is an imperative need for an enriched understanding of its influence on their psychological well-being and academic outcomes. For graduate students, the stakes are higher, given the demanding and intricate nature of their academic pursuits. Drigas and Papoutsi (2018) postulated that emotional education is crucial for cultivating essential social, cognitive, and emotional competencies, thereby becoming an integral component for the holistic development and thorough preparation of graduate students.

Existing research has yet to fully explore the interplay between emotional education and the psychological well-being of graduate students. A knowledge void persists regarding the impact of emotional education on managing stress, anxiety, and the unique emotional challenges encountered by this demographic. A nuanced exploration of how training in emotional education fosters the development of pertinent emotional and social skills, crucial for their academic and professional trajectories, is warranted.

Furthermore, emotional education is instrumental in enhancing the emotional well-being and mental health of graduate students. Housman (2017) contends that it is integral for honing emotional self-regulation skills, thereby positively influencing emotional well-being and mental health. In a strenuous academic milieu, proficiency in emotional education can equip graduate students with the tools to adeptly navigate negative emotions and cultivate resilience.

Addressing the research deficit in this domain underscores the necessity of a study exploring the correlation between emotional education and psychological well-being among graduate students. This exploration is pivotal for identifying and addressing the lacunae in the emotional education provided and for discerning avenues for enhancement in the graduate programs at Milagro State University. The study aims to pinpoint specific facets of emotional well-being and education requiring refinement to elevate the educational quality at this institution.

The impetus for this study stems from the imperative to bridge the research gaps concerning emotional education in graduate students. By delving deeper into the nexus between emotional education and psychological well-being, the study aims to forge robust strategies and programs to bolster student well-being and academic accomplishments.

Moreover, the study holds particular relevance for Milagro State University, aiming to spotlight areas warranting refinement in terms of emotional education within graduate programs. The insights gleaned will be instrumental in shaping the development and rollout of tailored workshops, courses, and initiatives addressing the emotional requisites of graduate students.

It is anticipated that the study will amplify awareness about the significance of emotional education in the higher education landscape. By underscoring the importance of emotional education for both personal well-being and academic and professional success, the study aspires to advocate for its incorporation into curricula and the professional development of educators and practitioners interacting with graduate students.

In today's scenario, the recognition of the importance of imparting emotional education to graduate students by Milagro State University is paramount. Accordingly, this research endeavors to scrutinize the relationship between emotional education and psychological well-being among graduate students, conducting a comparative analysis across diverse programs offered by the university. The ultimate objective is to identify the elements of emotional well-being and education that warrant enhancement, thereby optimizing the educational quality provided by the institution.

Additionally, given the diversity in participants, potential differences in outcomes related to sex may emerge in the study. Sex-specific distinctions in emotional education and psychological well-being could offer valuable insights, adding a nuanced layer to the research findings and contributing to the customization of interventions. By considering sex as a variable, the study aims to enrich the understanding of the interaction between emotional education and psychological well-being in the context of graduate students, thus enabling a more comprehensive and tailored approach to improving emotional education.

## Methods

The research presented in this document adheres to the positivist paradigm, which aims to uncover the true nature of reality through a quantitative approach ( Sparkes, 1992). This approach is grounded in a deductive and logical framework that allows for numerical measurement of variables and the utilization of descriptive statistics to address the research hypotheses. The unit of analysis comprises 1,522 randomly selected students, who are enrolled in various master's programs at Milagro State University.

To maintain an inclusive and comprehensive approach, the sex of the participants was defined based on self-identification. Participants were given the opportunity to identify themselves as male, female, or other, thereby recognizing the diversity and spectrum of sex identities. This approach is aligned with contemporary scientific and ethical standards, promoting inclusivity and respecting individual autonomy in self-identification.

For the collection of the necessary data, the Trait Meta-Mood Scale (TMMS-24) questionnaire, created by Salovey *et al.* (1995), was administered. This questionnaire consists of 24 items that assess individual differences in the ability to be aware of and regulate one's own emotions. Participants responded to the questionnaire using a 5-point Likert scale, where 1 equates to “Strongly Disagree” and 5 to “Strongly Agree”. It is worth noting that this instrument is open access, and is under a Creative Commons license, guaranteeing its availability and accessibility for future research and studies in the field of emotional regulation.

The scale is divided into three dimensions: emotional attention, emotional clarity, and emotional repair. In the process of assessing perceived emotional intelligence (PEI), a comprehensive numerical representation of each dimension is generated by aggregating the scores corresponding to each of the items that make up that dimension. The index of perceived emotional intelligence (PEI) is then computed by summing the total scores of the three dimensions, serving as a composite measure encapsulating the individual's perceived emotional capacity.

A reliability analysis is conducted for each dimension to ensure the internal consistency of the scales. Subsequently, descriptive statistics of the scores obtained in each dimension and the PEI were generated, offering a general overview of the data behavior. A correlation analysis was carried out to examine the specific relationships between the scores obtained in the different measurements and the PEI.

Lastly, an analysis of variance (ANOVA) was performed to investigate the impact of variables such as sex, age, and field of study on the dimensional scores and the PEI. This analysis aids in discerning if there are significant differences in scores based on these demographic and academic categories, thereby providing valuable insights into the influence of these factors on perceived emotional intelligence. The inclusion of sex as a variable, defined through self-identification, contributes to a nuanced understanding of its role and influence on the outcomes of the study, ensuring a robust and comprehensive exploration of the relationship between emotional education and psychological well-being.

### Ethical considerations

All participants in this study provided informed consent, in compliance with ethical standards for research involving human subjects. They were assured that their participation was voluntary and that they could withdraw from the study at any time without penalty. Confidentiality was maintained by anonymizing all personal data. The research was approved by the Institutional Review Board (IRB) at Milagro State University, under the approval “Oficio Nro. UNEMI-VICEINVYPOSG-DP-155-2023-OF,” dated February 13, 2023.

## Results and discussion

The evaluation of the PEI construct, as measured by the TMMS, is critical for understanding various psychological phenomena. The robustness and validity of such an instrument are therefore essential. First, the reliability of an instrument can be assessed through its internal consistency. When examining the dimensions of the TMMS, it's evident that Cronbach's alpha values are in a satisfactory range. The dimensions of Attention, Clarity, and Repair showed alphas of 0.87, 0.89, and 0.85, respectively. These values not only exceed the conventionally accepted threshold for good internal consistency but also closely resembled those found by Extremera and Fernández-Berrocal (2006). This parallel suggests the replicability and stability of the TMMS structure across different samples.

Based on the thorough analysis delineated in Table 1, a noteworthy trend was discerned in the scores across the dimensions of PEI. The confluence of minimum scores for Attention and Repair aligning with the lower limit of the scale invites multifaceted interpretations. For example, as postulated by Fiske (2018), this pattern could be indicative of a pronounced deficit in these particular emotional competencies among certain individuals, suggesting a reduced propensity to attend to or modulate emotions in challenging contexts.

**Table 1.** Descriptive statistics: minimum, range, mean, and standard deviation for the scores of the dimensions and PEI.

Dimension	Min	Range	Mean	SD
Attention	8.00	32.00	27.4987	6.06336
Clarity	11.00	29.00	31.1196	5.91481
Repair	8.00	32.00	31.9619	5.75433
PEI	39.00	81.00	90.58	13.94

Conversely, the scores in the Clarity dimension do not reach the lower extremity of the scale, intimating that even those with diminished emotional capacities retain some level of insight into their emotions. This finding is congruent with the proposition by Lee et al. (2020), positing that emotional clarity is a rudimentary competency, foundational for everyday functioning.

Analyzing the mean and dispersion revealed a congruent pattern between Clarity and Repair, inferring a potential interrelation. This correlation supports the theory by Extremera and Fernández-Berrocal (2005), which underscores the necessity of comprehending emotions (Clarity) as a precursor to effective management (Repair). In contrast, the Attention dimension manifested a broader distribution, indicative of the heterogeneity in individuals’ propensity to perceive and acknowledge their emotions, a variability echoed by Mauss and Robinson (2009).

Incorporating sex into the analysis offered an enriched perspective, enabling a nuanced exploration of how sex dimensions might interplay with individuals' emotional competencies. This incorporation is pivotal, shedding light on potential disparities or similarities in emotional intelligence across sex identities, thereby contributing to a more comprehensive understanding of the subject.

The examination of the interdimensional correlations of PEI unfolded patterns consistent with existing literature on emotional intelligence. The substantial correlation between Clarity and Repair (coefficient of 0.637) underscores the symbiotic relationship between the comprehension and modulation of emotions, aligning with insights from Haver et al. (2021).

Conversely, the subdued correlations between Attention and the other two dimensions might suggest that heightened awareness does not invariably lead to enhanced understanding or adept emotional regulation—a notion corroborated by Sekreter (2019).

Finally, the strong correlations across all dimensions with the overarching PEI substantiate the framework posited by Alzoubi and Aziz (2021), affirming the integral role each dimension plays in the holistic ability to perceive, comprehend, and regulate emotions. This implies that each dimension, while pivotal independently, also significantly informs and shapes the comprehensive construct of PEI.

Table 2, detailed below, offers a concise representation of these Pearson correlations between the TMMS dimensions and PEI. Each numerical value in the table reflects the strength and direction of the relationship between the dimensions, providing a solid foundation for deeper interpretations and future research in this area.

**Table 2.** Pearson Correlations.

	Attention	Clarity	Repair	PEI
Attention	1	0.369**	0.283**	0.708
	0.000	0.000	0.000	
Clarity		1	0.637**	0.847
			0.000	0.000
Repair			1	0.806
				0.000

Note: Correlations marked with ** are significant at the 0.01 level (two-tailed).

Within the sphere of scientific inquiry, elucidating the potential ramifications of diverse factors on principal variables is paramount. This research embarked on a nuanced exploration of how distinct elements, notably sex, age, and educational attainment, might modulate the dimensions of emotional intelligence—Attention, Clarity, and Repair—and the composite measure of PEI.

The discernible variance in the relationship between these elements and the PEI dimensions within the context of the TMMS necessitates scrupulous scrutiny. The manifestation of sex, age, and educational attainment as independent variables substantiates the concept that individual and demographic attributes engage in intricate interplays with emotional intelligence, resonating with earlier studies ( Deng, 2021; Manca *et al.,* 2019).

In particular, the significant influence of age and educational attainment on the Attention and Clarity dimensions aligns with the extant literature. The evolution of emotional maturity, concomitant with aging, can potentially enhance the faculties to discern and clarify emotions ( Gao *et al*., 2021; Vaish *et al.,* 2008). Concurrently, educational attainment, emblematic of proficiency and aptitude in a particular field, could furnish a structured paradigm, thereby facilitating the processes of emotional discernment and clarification.

The integration of sex as a variable in this discourse enriches the analysis, offering a lens through which to examine its interaction with emotional intelligence dimensions. This integration is pivotal as it delves into the nuances of how sex may uniquely contribute to the dynamics of emotional intelligence, thereby augmenting the comprehensiveness of the study.

Interestingly, the singular impact of age on the Repair dimension prompts contemplation, positing that the capability to amend and regulate emotions might be more contingent upon accrued experience and maturity than on elements such as proficiency in a particular domain. This observation presents a divergence from the findings of Herd and Kim-Spoon (2021), who elucidated the progressive enhancement and adaptation of emotional regulation through time and experience.

Moreover, the salience of educational attainment as the sole significant determinant in the overarching measure of PEI underscores the pivotal role of domain-specific competence and expertise in shaping the holistic perception of emotional intelligence. This inference aligns with the conceptualization by Dolev and Leshem (2017), proposing that the manifestation of emotional intelligence in academic and professional realms is predominantly influenced by competence within those domains.

These results underline the complexity and specificity of the interactions between the dimensions of emotional intelligence and the demographic and academic factors under study. For a more detailed and visual understanding of these findings, Table 3 provides a comprehensive breakdown of the variance analysis performed.

**Table 3.** Sum of squares for the sources of variation in the analysis of variance model.

Origin	df	Attention	Clarity	Repair	PEI
Corrected Model	6	1073.22	838.67	438.74	3693.22
Intercept	1	229960.87	275653.11	294560.43	2394147.66
Sex	1	3.44	14.91	3.10	.059
Age	3	568.87*	584.56*	344.07*	1472.74
Mastery	2	541.39*	207.15*	97.280	2210.82*
Error	1511	59310.53	52230.28	49873.11	309081.72
Total	1,518	1,274,641.00	1,522,426.00	1,600,331.00	12,979,978.00
Corrected Total	1,517	60,383.76	53,068.962	50,311.864	312,774.954

The multifaceted nature of emotional intelligence, illuminated by a plethora of studies examining it from diversified vantage points, accentuates the imperative of identifying underlying patterns within the amassed data ( Azevedo *et al*., 2013; Gooty *et al.,* 2014). This present investigation meticulously underscores the capacity to extract such nuanced patterns, thereby offering an intricate framework elucidating the interplay between certain demographic, academic factors, and distinctive dimensions of emotional intelligence.

The study's results, particularly those relating to the “attention” dimension, unveil a noteworthy convergence between students in the 21 to 30 years age bracket and those surpassing 50 years. Such a pattern potentially alludes to the existence of distinct life phases where attentiveness to emotions experiences amplification. Dickerson and Quas (2021) have theorized that emotional awareness might be particularly amplified both in youth—attributed to the transitions and revelations inherent to this life stage—and in older age, characterized by heightened reflection and self-awareness.

Delving into the dimensions of “clarity” and “repair,” the ascension in mean scores concurrent with aging intimates an enhancement associated with accrued life experiences and the refinement of emotional regulation competencies—a proposition corroborated by preceding research ( Blasco-Belled *et al*., 2020; Martínez-Marín *et al*., 2021). Concurrently, the prominent scores of Early Education students in pivotal dimensions of emotional intelligence may be ascribed to the inherent characteristics of their scholastic curriculum. Education, predominantly at the foundational level, invariably necessitates profound empathy and refined interpersonal acumen ( Blakemore and Agllias, 2020). The cultivation and comprehension of emotions, both personal and of others, are indispensable in this field, thereby potentially manifesting in the elevated scores observed among these students.

Moreover, incorporating sex as an analytical variable in this discourse augments the depth of the study, furnishing a nuanced perspective to discern its potential interactions with the facets of emotional intelligence. This inclusion is quintessential, elucidating the intricate ways in which sex may shape and influence the dynamics of emotional intelligence, thereby enriching the holistic understanding of this complex construct. The intersectionality of sex with other demographic and academic variables provides a fertile ground for further exploration, potentially unveiling additional layers of complexity and contributing to a more comprehensive and nuanced appreciation of emotional intelligence in diverse populations.

Table 4 provides a detailed analysis of the interaction between demographic and academic variables with the dimensions of emotional intelligence. Through the DMS Test for mean comparison, significant differences and similarities were determined among the groups. The assigned letters (A, B, C) denote the statistical significance between the means; means with the same letter show no significant differences at the 0.05 level.

**Table 4.** DMS analysis of mean comparison for age and mastery variables.

Factors and ranges	Attention	Clarity	Repair	IEP
Age (years)				
21 to 30	29.40 A1	29.87 C	31.04 C	90.33 A
31 to 40	27.92 B	30.55 BC	31.79 C	90.26 A
41 to 50	27.33 B	31.18 AB	32.20 AB	90.72 A
Over 50	28.32 AB	32.07 A	32.82 A	93.21 A
Mastery				
Education	26.79 B	30.26 B	31.65 A	88.71 B
Basic education	28.37 B	30.84 B	31.82 A	91.04 B
Early education	29.56 A	31.66 A	32.41 A	93.64 A

Note: Means with the same letter (A, B, C) in each column indicate that there are no statistically significant differences between them, according to the DMS Test criteria at a significance level of p<0.05.

A pivotal insight emanating from the conducted analysis pertains to the substantial correlation between emotional clarity and age. The existing corpus of research has consistently underscored the evolution of the capacity to interpret and comprehend emotions, a progression modulated by a confluence of biological underpinnings and accrued socio-emotional learning ( Averill, 1980; Mayer *et al*., 2016). The Chi-squared value of 22.27 manifested in this investigation fortifies this conceptualization, emphasizing that emotional clarity is a dynamic attribute, subject to vicissitudes across the chronological trajectory of an individual's existence.

Concurrently, the discerned disparities in the dimensions of attention and clarity associated with sex, substantiated by Chi-squared values of 53.96 and 18.83, cohere with the existing scholarly discourse. This body of literature posits that variations exist in the ways men and women process and engage with emotions, potentially attributable to a matrix of sociocultural constructs and inherent biological determinants ( Averill, 1976; Meyers-Levy and Loken, 2015; Whittle *et al*., 2017). It is imperative to elucidate that such differences do not insinuate a hierarchical dichotomy or superiority of one over the other; rather, they signify diversification in the modalities of experiencing and conceptualizing emotions.

Furthermore, the discernible association between one's field of mastery and the dimensions of emotional intelligence, corroborated by Chi-squared values of 13.34 and 9.93, intimates the influential role of academic training and experiential learning in sculpting emotional perception and discernment. It emerges as plausible that distinct academic disciplines, by virtue of their inherent characteristics and thematic emphasis, foster competencies germane to emotional recognition and clarity ( Chen and Lu, 2022; Denham and Brown, 2010).

**Figure 1.**
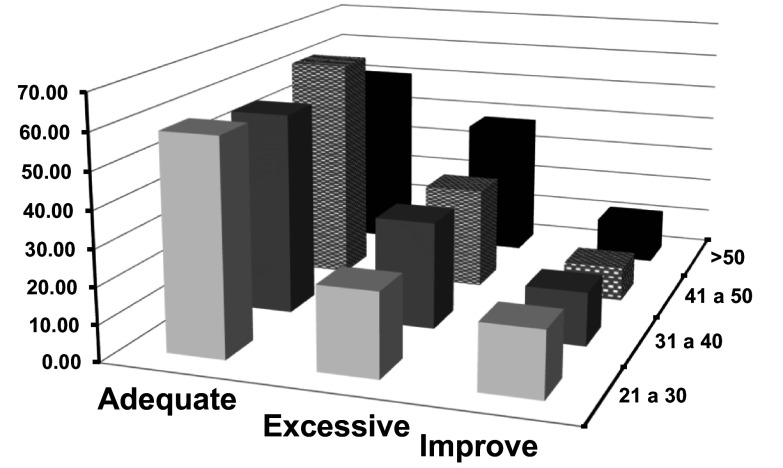
Frequencies of categorized clarity crossed with age.

Figure 1, presented below, illustrates this interaction. It provides a visual representation of the frequencies of categorized clarity according to different age cohorts. This type of graphical representation allows for a more intuitive interpretation of the data, highlighting trends, consistencies, or possible anomalies among the groups.

The graphical representation in Figure 1 highlights these temporal dynamics of emotional perception in relation to age. It is revealing to observe that, with advancing age, there is an increase in the “excessive” category. This trend could align with the idea that accumulated experiences and introspection throughout life enrich emotional perception ( Bukor, 2015). Older individuals may have a more finely-tuned ability to recognize and understand the depth and complexity of their emotions, resulting in an “excessive” perception of emotional clarity.

In contrast, the “improve” category exhibits a notable decline with age. This could be interpreted as increased confidence and certainty in emotional perception acquired over the years. The fact that this trend starts high in the youngest group and decreases with age suggests that, over time and experience, there is a consolidation of emotional understanding ( Casale *et al.*, 2022).

However, while these trends provide a fascinating insight into the evolution of emotional clarity throughout life, it is essential, as in all research, to maintain a critical perspective. Individual differences, cultural factors, socioeconomic factors, and life experiences can modulate these patterns ( Gross and John, 1995; Seixas *et al.*, 2021).

**Figure 2.**
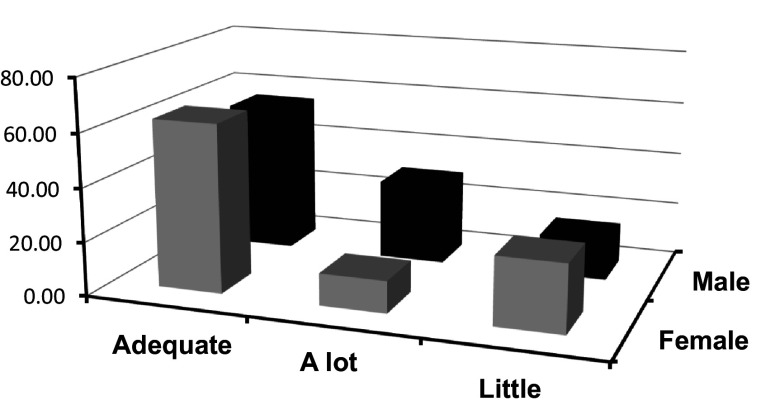
Frequencies of categorized attention crossed with sex.

Next, Figure 2 is presented, where a visual representation of the frequencies of categorized emotional attention based on sex is displayed. This graph provides a revealing perspective on how sex differences may be associated with variations in attention directed towards emotions.

In Figure 2, which illustrates the distribution of attention frequencies according to sex, distinctive patterns are observed in relation to the categories of emotional attention, mainly in the “adequate,” “little,” and “much” categories.

The female sex appears to predominate in the “adequate” and “little” categories, suggesting that females, in this study, tended to report levels of emotional attention that range between moderate and low. On the other hand, in the “much” category, a predominance of the male sex was observed, indicating that men in this sample report a high level of attention towards their own emotions or those of others.

These observations are consistent with literature in Psychology and Neuroscience that has explored sex differences in emotional perception and regulation. For example, Kashdan *et al*. (2009) posited that, in general, women may be more attuned to emotions, which could translate into more balanced or “adequate” levels of emotional attention. However, this does not necessarily imply that women pay less attention to emotions than men. Instead, it suggests that women may be more efficient in regulating the amount of attention they give to emotions, avoiding extremes.

On the other hand, the fact that men stand out in the “much” category could reflect a tendency among some men to be hyper-aware or excessively attentive to certain emotions. This pattern could be related to sociocultural norms that, in many contexts, discourage emotional expression among men, leading them to greater introspection and self-awareness ( Dworkin and Weaver, 2021).

However, it is crucial not to generalize these findings to all populations or interpret them as reflecting fixed biological differences between sex The observed differences may be influenced by sociocultural, educational factors, and individual life experiences.

**Figure 3.**
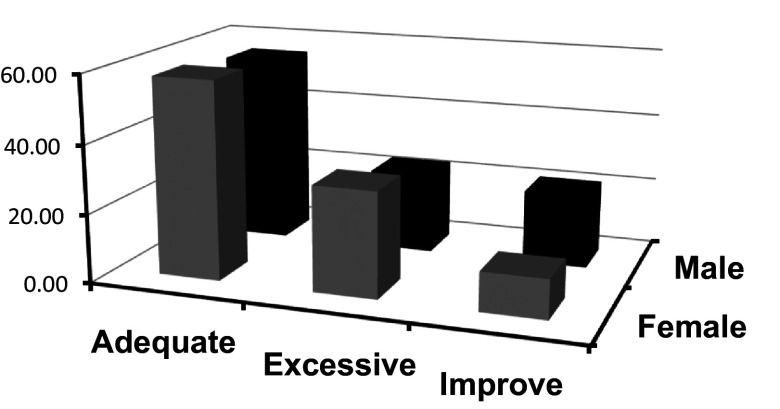
Frequencies of categorized emotional clarity crossed with sex.

In the following Figure 3, it is illustrated how the frequencies of categorized emotional clarity vary according to sex. This visualization will allow the exploration of whether there are distinctive patterns between sex concerning the clarity of their emotions.

In the field of emotional intelligence, emotional clarity denotes an individual's ability to identify and understand their own emotions, an aspect that is of great importance as it provides insights into how people process and manage their emotions in daily life. Figure 3 offers a detailed view of the frequencies of categorized emotional clarity according to sex.

According to the data presented in Figure 3, it is observed that women tend to report higher frequencies of levels of emotional clarity considered “adequate” or “excessive” compared to men. However, in the “improve” category, men surpass women in frequency, suggesting a greater inclination on their part to feel that they need to improve in this aspect.

These differences can be interpreted from various perspectives. According to Doherty (1997), women tend to be more introspective and are socially conditioned to be more expressive and aware of their emotions, which could explain the higher frequencies in the “adequate” and “excessive” categories observed in females. In contrast, men, subject to certain sociocultural norms, might feel less encouraged to explore and understand their emotions, which could influence their perception of the need to “improve” in this area.

Additionally, it is interesting to highlight the hierarchy observed in the frequencies for both categories: “adequate”, “excessive”, and “improve”. These findings underline that, regardless of sex, most individuals feel that they possess an acceptable degree of emotional clarity. However, it is in the “improve” category where the most significant contrast between the sexes is found, prompting reflections on the differences in emotional self-assessment and the potential sociocultural influences underlying this perception.

Finally, it is imperative to emphasize that, while these findings are revealing, they should not be interpreted simplistically. Differences in the self-perception of emotional clarity can be multifactorial and, as suggested by Gomez-Baya *et al*. (2017), may be influenced by both biological and sociocultural factors. In this context, a detailed understanding of the observed patterns related to emotional clarity and sex opens doors for future research and reflections in the field of Emotional Psychology.

**Figure 4.**
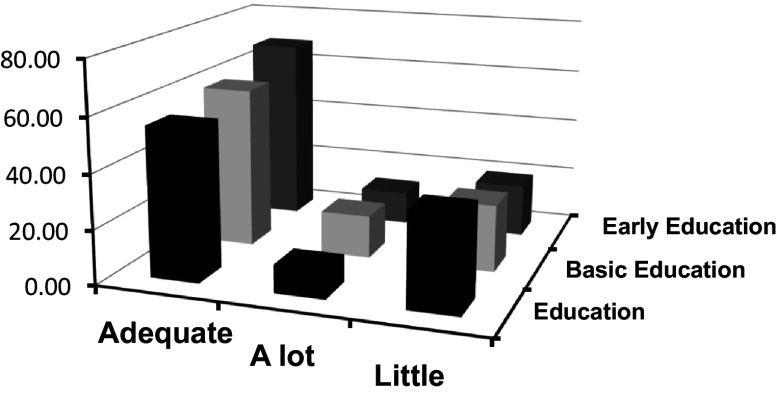
Frequencies of categorized clarity crossed with type of Master's program.

The following Figure 4 offers a revealing perspective on the interaction between emotional clarity and academic specialization at the Master's level. Through this graphic representation, the aim is to analyze the possible influence of academic training on the perception and understanding of emotions among postgraduate students.

Emotional attention refers to an individual's ability to tune into and be aware of their own emotions as well as those of others. This construct has crucial implications in the academic and professional world, as the way an individual pays attention to emotions can influence their learning and teaching abilities, as well as their capacity to interact with others in educational contexts.

According to Figure 4, a notable relationship is evident between attention categories and the type of Master's program students are enrolled in. This relationship provides information on how different specializations may influence students' self-perception of emotional attention.

A key finding observed is the gradient in the “adequate” category. Parada-Cabaleiro *et al*. (2020) argued that adequate perception of emotions is crucial for effective emotional processing. Students in "Early Childhood Education," who often work with young children, might develop more finely-tuned emotional attention due to the intuitive and non-verbal nature of communication at early ages. This finding is corroborated by results obtained in studies like that of Joss *et al.* (2020), who argue that working with younger populations can cultivate greater sensitivity and attention to emotions.

On the other hand, the “low” category suggests a perception of insufficient emotional attention. Here, the highest frequency corresponds to students in the "Education" Master's program. According to Jamil *et al.* (2023), traditional education often prioritizes cognitive skills over emotional skills. However, it is essential to consider that the type of population or educational context these programs target may influence these results.

Lastly, the “high” category presents an interesting pattern. The "Basic Education" Master's holds the highest point, indicating that these students perceive elevated emotional attention. Aguado *et al.* (2018) argue that emotional attention can be influenced by the demands of the context. Basic education often requires a greater capacity to tune into a wide range of emotions due to the diversity of ages and issues.

**Figure 5.**
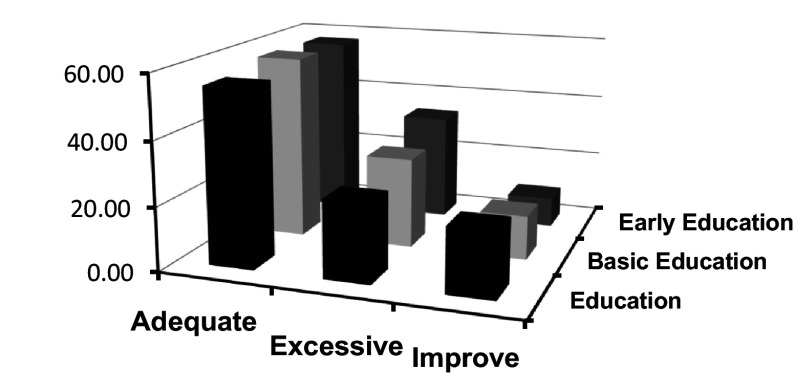
Frequencies of categorized clarity crossed with type of Master's program.

In Figure 5, the distribution of frequency categories of clarity, which have been crossed with different types of Master's programs, is illustrated. This graphic representation aims to establish a relationship between the perception of clarity and different postgraduate students, allowing for a more detailed evaluation and a deeper interpretation of the collected data.

Figure 5 offers a detailed view of how emotional clarity relates to the academic specialization that students choose at the Master's level. This graphical representation indicates a notable dependency between the perception of emotional clarity and the nature of the academic program being pursued.

Analysis of the graph reveals that the categories of “adequate” and “excessive” in emotional clarity exhibit a pattern of consecutive increase from students in "Education" to those in "Early Childhood Education" ("Ed. Inicial" in the original text). This finding is consistent with previous research that suggests that certain academic programs, especially those oriented towards initial training in education, may place a stronger emphasis on emotional development ( Hodson, 2003; McCoy and Wolf, 2018).

On the other hand, it is intriguing to note that the “improve” category reflects an inverse trend. Specifically, those students in the "Education" Master's program show higher percentages, which could imply a greater awareness of their areas for improvement in emotional understanding or, alternatively, a program that has yet to fully emphasize this domain, as suggested by some authors ( Yang *et al.*, 2022).

It is essential to point out that while these patterns may reflect the influence of curriculum and pedagogy on the perception of emotional clarity, they could also possibly reflect preexisting characteristics of the students who choose these programs. For example, those with intrinsically high emotional clarity may be more attracted to programs like "Early Childhood Education," given their direct and profound interaction with child development ( Ryan and Deci, 2020).

## Conclusions

Initiating a study that seeks to decipher the relationship between demographic and emotional variables represents a challenge. However, the results of this research shed light on this complex interaction, with a specific focus on emotional clarity and attention. Based on rigorous quantitative methodology, detailed relationships were discovered that model these emotional capacities in various populations.

The relationship evidenced between age and emotional clarity is undeniable. As the years pass, lived experiences and maturity have a positive impact on people's interpretation and response to their emotions. This relationship not only reinforces traditional concepts about the wisdom associated with aging but also offers opportunities, especially in tailoring interventions according to age groups for better outcomes.

On the other hand, when considering gender, differences were found in emotional attention and clarity, which are not only notable but also enlightening. It could be deduced that these differences arise from a combination of factors, from socio-cultural constructs to biological differences. Understanding these variations is essential, and from this perspective, it becomes imperative to reconsider and adapt intervention and education strategies for an equitable approach.

Following this line of research, the link between academic training and emotionality also stood out, particularly regarding Master's levels. It was revealed that certain academic specializations play a significant role in the development of people's introspection and emotional awareness. This relationship strengthens the bond between formal education and emotional skills.

In light of this revelation, it would be appropriate to consider that the adaptation and refinement of specific pedagogical methods could strengthen emotional clarity and attention according to the academic realm. This adaptation in teaching provides a scenario where future professionals could develop with greater emotional acumen and self-awareness.

However, it is essential to take into account the practical implications of these findings. It is evident that any educational or therapeutic planning must consider these demographic variables. Ignoring these differences carries the risk of implementing less effective or incorrect interventions.

Emphasizing this aspect, it is essential for professionals in education, therapy, and mental health to be aware of these discoveries. With this information, they can design and apply strategies tailored to individual needs, enhancing their beneficial impact.

Although this work contributes to the academic field, it should not be considered as a definitive answer. It should be viewed more as a foundation for future research. The dynamics between demography and emotional skills have multiple facets, and it is likely that many aspects remain to be explored.

From this perspective, the study does not end a debate, but invites future research. The questions that arise, particularly regarding how other socio-economic or demographic factors might influence emotional clarity and attention, have great potential. This work aims to encourage future explorations in an area of such relevance and fascination.

## Data Availability

Figshare: Data-Comparative Analysis of Psychological Well-being.xlsx. https://doi.org/10.6084/m9.figshare.24148155.v1 The project contains the following underlying data:
- Data-Comparative Analysis of Psychological Well-being.xlsx Data are available under the terms of the Creative Commons Attribution 4.0 International license (CC-BY 4.0).
