# Comparative Analysis of Psychological Well-Being and Emotional Education in Graduate Students

**DOI:** 10.12688/f1000research.141849.2

**Published:** 2024-08-06

**Authors:** Jenniffer Sobeida Moreira-Choez, Tibisay Milene Lamus de Rodríguez, Eduardo Javier Espinoza-Solís, Graciela Josefina Castro-Castillo

**Affiliations:** 1 Posgrado, State University of Milagro, Milagro, Guayas, 091706, Ecuador

**Keywords:** Emotional intelligence, psychological well-being, emotional education, educational strategies, graduate students

## Abstract

**Background:**

The growing importance of emotional intelligence in academic and professional contexts has generated a need to explore its linkage with psychological well-being. Furthermore, understanding how various demographic and academic factors can influence students' emotional perception and management is crucial for optimizing educational and intervention strategies. In this context, the primary purpose of this study was to analyze the existing relationship between emotional education and psychological well-being in graduate students.

**Methods:**

The objective was to conduct a comparative analysis of perceived emotional intelligence (PEI) in different study programs offered at a specific university. The methodology, framed within a positivist paradigm, was based on a quantitative approach and examines the responses of 1,522 university students using the Trait Meta-Mood Scale (TMMS-24).

**Results:**

This tool, which is divided into three dimensions (emotional attention, emotional clarity, and emotional repair), was analyzed using descriptive statistics, correlation analysis, and ANOVA tests to determine demographic and academic influences on the scores. The findings indicate deficiencies in the areas of Emotional Attention and Emotional Repair, contrasting with a marked prevalence in Emotional Clarity. Variables such as sex, age, and field of study demonstrated an influence on the dimensions of PEI. Notably, significant differences in emotional perception were found between sex and academic fields.

**Conclusions:**

Specifically, training directed towards empathy proved to be a prominent factor in the perception of emotional competencies. This study highlights the influence of demographic and academic variables on emotional competencies, underscoring the need to adapt strategies in education and therapy.

## Introduction

In recent years, the significance of emotional education has increasingly been recognized across various societal sectors due to its essential role in personal and professional development. Defined by Zhang and Adegbola (2022) as the ability to identify, comprehend, and manage both one’s own and others’ emotions, emotional education is crucial for fostering healthy interpersonal relationships and effectively managing complex emotional situations.

Despite the heightened attention it has received, substantial research gaps remain, particularly concerning the effects of emotional education on graduate students. These students encounter unique pressures that make understanding the impact of emotional education on their psychological well-being and academic success particularly pertinent ( Boekaerts, 1993; Hammoudi Halat *et al*., 2023). Drigas and Papoutsi (2018) argue that emotional education is indispensable for developing key social, cognitive, and emotional competencies, integral for the comprehensive development and thorough preparation of graduate students.

The existing body of research has not adequately addressed how emotional education influences the psychological well-being of graduate students, nor has it explored its role in managing stress, anxiety, and the distinct emotional challenges faced by this group. There is a clear need for detailed investigation into how emotional education contributes to developing relevant emotional and social skills that are critical for their academic and professional paths.

Moreover, emotional education plays a key role in improving the emotional well-being and mental health of students ( Fitzgerald *et al*., 2022; Yorke *et al*., 2021). Housman (2017) emphasizes that it is essential for refining emotional self-regulation skills, which positively impact mental health and emotional stability. In the demanding academic environment that graduate students navigate, proficiency in emotional education can provide them with the necessary tools to manage negative emotions effectively and foster resilience ( Bernstein *et al*., 2021; Loewenberg, 1969; Malandraki, 2022).

Recognizing these issues underscores the need for comprehensive research examining the relationship between emotional education and psychological well-being among graduate students ( Iwamoto & Liu, 2010; Prakash *et al.*, 2023). This investigation is crucial for identifying deficiencies in the current educational offerings and for exploring ways to enhance the graduate programs at Milagro State University. The study aims to identify specific aspects of emotional well-being and education that require improvement to elevate the quality of education provided at this institution.

The motivation for this study is rooted in the urgent need to address these gaps in research on emotional education among graduate students. By exploring the connection between emotional education and psychological well-being, the study seeks to develop effective strategies and programs that enhance student well-being and academic performance ( Yamin *et al*., 2023).

This research is of particular importance to Milagro State University as it seeks to identify and improve areas of emotional education within its graduate programs. The insights obtained are expected to guide the creation and implementation of customized workshops, courses, and initiatives that meet the emotional needs of graduate students.

It is anticipated that the study will significantly increase awareness of the importance of emotional education in the higher education landscape. Highlighting the benefits of emotional education for both personal well-being and academic and professional success, the study advocates for its integration into curricula and the professional development of educators and practitioners working with graduate students.

In the current educational climate, the commitment of Milagro State University to enhancing emotional education for graduate students is paramount. This research will conduct a thorough examination of the relationship between emotional education and psychological well-being across various graduate programs, aiming to pinpoint elements that need enhancement to optimize the educational quality offered.

Additionally, given the diversity in participants, potential differences in outcomes related to sex may emerge in the study. Sex-specific distinctions in emotional education and psychological well-being could offer valuable insights, adding a nuanced layer to the research findings and contributing to the customization of interventions. By considering sex as a variable, the study aims to enrich the understanding of the interaction between emotional education and psychological well-being in the context of graduate students, thus enabling a more comprehensive and tailored approach to improving emotional education.

## Methods

The current research adhered to a quantitative approach within the positivist paradigm, aiming to unravel the structure of reality through systematic and deductive analysis of quantifiable variables he researches presented in this document adheres to the positivist paradigm, which aims to uncover the true nature of reality through a quantitative approach ( Sparkes, 1992). The sample included 1,522 students from master’s programs in Education, Basic Education, and Early Education at Milagro State University. The sample size was established using a statistical power calculation to ensure the ability to detect significant effects, setting a confidence level of 95% and a power of 80%.

Regarding demographics, the identification of participants’ sex was conducted through self-identification, allowing them to be categorized as male, female, or other. This methodology aligns with contemporary scientific and ethical standards, promoting inclusivity and respect for personal autonomy.

For data collection, the Trait Meta-Mood Scale (TMMS-24) by Salovey *et al.* (1995), was used, which assesses emotional regulation and awareness through 24 items. Participants responded using a five-point Likert scale. This instrument is available under a Creative Commons license, which facilitates its use in future research.

The reliability analysis of the TMMS-24 scales yielded Cronbach’s alpha coefficients of 0.87, 0.89, and 0.85 for the Emotional Attention, Clarity, and Repair dimensions, respectively. These values, indicating robust internal consistency, are comparable to those reported by Extremera and Fernández-Berrocal (2005), suggesting the structural stability of the TMMS across different samples.

Descriptive statistics were generated for each dimension of the TMMS-24 and Perceived Emotional Intelligence (PEI), including range, minimum, mean, and standard deviation, providing an overview of the behavior of the variables (see Table 1). Subsequently, correlation and variance analyses (ANOVA) were conducted to examine the impact of demographic and academic variables such as sex, age, and field of study on the scores. The ANOVA analyses included Tukey post hoc tests when significant differences were detected, ensuring an appropriate comparison between groups. Additionally, the normality and homogeneity of variances were verified to validate the assumptions of ANOVA, using the Shapiro-Wilk and Levene tests, respectively.

**Table 1.** Descriptive statistics: minimum, range, mean, and standard deviation for the scores of the dimensions and PEI.

Dimension	Min	Range	Mean	SD
Attention	8.00	32.00	27.4987	6.06336
Clarity	11.00	29.00	31.1196	5.91481
Repair	8.00	32.00	31.9619	5.75433
PEI	39.00	81.00	90.58	13.94

The interdimensional analyses of the PEI revealed significant correlations, validating previous theories about the relationship between understanding and regulating emotions. These findings, supported by correlation coefficients and significance levels, provide a solid foundation for deeper interpretations and future research in this field (see Table 2).

**Table 2.** Pearson Correlations.

	Attention	Clarity	Repair	PEI
Attention	1	0.369**	0.283**	0.708
	0.000	0.000	0.000	
Clarity		1	0.637**	0.847
			0.000	0.000
Repair			1	0.806
				0.000

Note: Correlations marked with ** are significant at the 0.01 level (two-tailed).

Finally, the integration of variables such as sex into the analysis provided an enriched perspective and allowed for a detailed exploration of how these dimensions interact with individuals’ emotional competencies, thus contributing to a broader and deeper understanding of the phenomenon studied.

### Ethical considerations

In the current study, all participants provided informed consent, thereby complying with the ethical standards established for research involving human subjects. They were assured that their participation was completely voluntary and that they were free to withdraw from the study at any time without any penalty. To ensure confidentiality, all personal data were anonymized. This research process received approval from the Institutional Review Board (IRB) of Milagro State University, under official document number UNEMI-VICEINVYPOSG-DP-155-2023-OF, dated February 13, 2023.

To ensure rigor in the development and presentation of research reports, specific guidelines that promote transparency and replicability of the study were followed. This methodological approach allows other researchers to evaluate and replicate the obtained results, which in turn contributes to the integrity and reliability of quantitative research. In this context, standardized protocols for data collection and analysis were adopted, thus ensuring objectivity and minimizing potential biases in the interpretation of the results.

### Results and discussion

The evaluation of the PEI construct, as measured by the TMMS, is critical for understanding various psychological phenomena. The robustness and validity of such an instrument are therefore essential. First, the reliability of an instrument can be assessed through its internal consistency. When examining the dimensions of the TMMS, it’s evident that Cronbach’s alpha values are in a satisfactory range. The dimensions of Attention, Clarity, and Repair showed alphas of 0.87, 0.89, and 0.85, respectively. These values not only exceed the conventionally accepted threshold for good internal consistency but also closely resembled those found by Extremera and Fernández-Berrocal (2005). This parallel suggests the replicability and stability of the TMMS structure across different samples.

Based on the thorough analysis delineated in Table 1, a noteworthy trend was discerned in the scores across the dimensions of PEI. The confluence of minimum scores for Attention and Repair aligning with the lower limit of the scale invites multifaceted interpretations. For example, as postulated by Fiske (2018), this pattern could be indicative of a pronounced deficit in these particular emotional competencies among certain individuals, suggesting a reduced propensity to attend to or modulate emotions in challenging contexts.

Conversely, the scores in the Clarity dimension do not reach the lower extremity of the scale, intimating that even those with diminished emotional capacities retain some level of insight into their emotions. This finding is congruent with the proposition by Lee *et al*. (2020), positing that emotional clarity is a rudimentary competency, foundational for everyday functioning.

Analyzing the mean and dispersion revealed a congruent pattern between Clarity and Repair, inferring a potential interrelation. This correlation supports the theory by Extremera and Fernández-Berrocal (2005), which underscores the necessity of comprehending emotions (Clarity) as a precursor to effective management (Repair). In contrast, the Attention dimension manifested a broader distribution, indicative of the heterogeneity in individuals’ propensity to perceive and acknowledge their emotions, a variability echoed by Mauss and Robinson (2009).

Incorporating sex into the analysis offered an enriched perspective, enabling a nuanced exploration of how sex dimensions might interplay with individuals’ emotional competencies. This incorporation is pivotal, shedding light on potential disparities or similarities in emotional intelligence across sex identities, thereby contributing to a more comprehensive understanding of the subject.

The examination of the interdimensional correlations of PEI unfolded patterns consistent with existing literature on emotional intelligence. The substantial correlation between Clarity and Repair (coefficient of 0.637) underscores the symbiotic relationship between the comprehension and modulation of emotions, aligning with insights from Haver *et al*. (2021).

Conversely, the subdued correlations between Attention and the other two dimensions might suggest that heightened awareness does not invariably lead to enhanced understanding or adept emotional regulation a notion corroborated by Sekreter (2019).

Finally, the strong correlations across all dimensions with the overarching PEI substantiate the framework posited by Alzoubi and Aziz (2021), affirming the integral role each dimension plays in the holistic ability to perceive, comprehend, and regulate emotions. This implies that each dimension, while pivotal independently, also significantly informs and shapes the comprehensive construct of PEI.

Table 2, detailed below, offers a concise representation of these Pearson correlations between the TMMS dimensions and PEI. Each numerical value in the table reflects the strength and direction of the relationship between the dimensions, providing a solid foundation for deeper interpretations and future research in this area.

Within the sphere of scientific inquiry, elucidating the potential ramifications of diverse factors on principal variables is paramount. This research embarked on a nuanced exploration of how distinct elements, notably sex, age, and educational attainment, might modulate the dimensions of emotional intelligence Attention, Clarity, and Repair and the composite measure of PEI.

The discernible variance in the relationship between these elements and the PEI dimensions within the context of the TMMS necessitates scrupulous scrutiny. The manifestation of sex, age, and educational attainment as independent variables substantiates the concept that individual and demographic attributes engage in intricate interplays with emotional intelligence, resonating with earlier studies ( Deng, 2021; Manca *et al.,* 2019).

In particular, the significant influence of age and educational attainment on the Attention and Clarity dimensions aligns with the extant literature. The evolution of emotional maturity, concomitant with aging, can potentially enhance the faculties to discern and clarify emotions ( Gao *et al*., 2021; Vaish *et al*., 2008). Concurrently, educational attainment, emblematic of proficiency and aptitude in a particular field, could furnish a structured paradigm, thereby facilitating the processes of emotional discernment and clarification.

The integration of sex as a variable in this discourse enriches the analysis, offering a lens through which to examine its interaction with emotional intelligence dimensions. This integration is pivotal as it delves into the nuances of how sex may uniquely contribute to the dynamics of emotional intelligence, thereby augmenting the comprehensiveness of the study.

Interestingly, the singular impact of age on the Repair dimension prompts contemplation, positing that the capability to amend and regulate emotions might be more contingent upon accrued experience and maturity than on elements such as proficiency in a particular domain. This observation presents a divergence from the findings of Herd and Kim-Spoon (2021), who elucidated the progressive enhancement and adaptation of emotional regulation through time and experience.

Moreover, the salience of educational attainment as the sole significant determinant in the overarching measure of PEI underscores the pivotal role of domain-specific competence and expertise in shaping the holistic perception of emotional intelligence. This inference aligns with the conceptualization by Dolev and Leshem (2017), proposing that the manifestation of emotional intelligence in academic and professional realms is predominantly influenced by competence within those domains.

These results underline the complexity and specificity of the interactions between the dimensions of emotional intelligence and the demographic and academic factors under study. For a more detailed and visual understanding of these findings, Table 3 provides a comprehensive breakdown of the variance analysis performed.

**Table 3.** Sum of squares for the sources of variation in the analysis of variance model.

Origin	df	Attention	Clarity	Repair	PEI
Corrected Model	6	1073.22	838.67	438.74	3693.22
Intercept	1	229960.87	275653.11	294560.43	2394147.66
Sex	1	3.44	14.91	3.10	.059
Age	3	568.87*	584.56*	344.07*	1472.74
Mastery	2	541.39*	207.15*	97.280	2210.82*
Error	1511	59310.53	52230.28	49873.11	309081.72
Total	1,518	1,274,641.00	1,522,426.00	1,600,331.00	12,979,978.00
Corrected Total	1,517	60,383.76	53,068.962	50,311.864	312,774.954

The multifaceted nature of emotional intelligence, illuminated by a plethora of studies examining it from diversified vantage points, accentuates the imperative of identifying underlying patterns within the amassed data ( Azevedo *et al*., 2013; Gooty *et* al., 2014). This present investigation meticulously underscores the capacity to extract such nuanced patterns, thereby offering an intricate framework elucidating the interplay between certain demographic, academic factors, and distinctive dimensions of emotional intelligence.

The study’s results, particularly those relating to the “attention” dimension, unveil a noteworthy convergence between students in the 21 to 30 years age bracket and those surpassing 50 years. Such a pattern potentially alludes to the existence of distinct life phases where attentiveness to emotions experiences amplification. Dickerson and Quas (2021) have theorized that emotional awareness might be particularly amplified both in youth attributed to the transitions and revelations inherent to this life stage and in older age, characterized by heightened reflection and self-awareness.

Delving into the dimensions of “clarity” and “repair,” the ascension in mean scores concurrent with aging intimates an enhancement associated with accrued life experiences and the refinement of emotional regulation competencies—a proposition corroborated by preceding research ( Blasco-Belled *et al*., 2020; Martínez-Marín *et al*., 2021). Concurrently, the prominent scores of Early Education students in pivotal dimensions of emotional intelligence may be ascribed to the inherent characteristics of their scholastic curriculum. Education, predominantly at the foundational level, invariably necessitates profound empathy and refined interpersonal acumen ( Blakemore and Agllias, 2020). The cultivation and comprehension of emotions, both personal and of others, are indispensable in this field, thereby potentially manifesting in the elevated scores observed among these students.

Moreover, incorporating sex as an analytical variable in this discourse augments the depth of the study, furnishing a nuanced perspective to discern its potential interactions with the facets of emotional intelligence. This inclusion is quintessential, elucidating the intricate ways in which sex may shape and influence the dynamics of emotional intelligence, thereby enriching the holistic understanding of this complex construct. The intersectionality of sex with other demographic and academic variables provides a fertile ground for further exploration, potentially unveiling additional layers of complexity and contributing to a more comprehensive and nuanced appreciation of emotional intelligence in diverse populations.

Table 4 provides a detailed analysis of the interaction between demographic and academic variables with the dimensions of emotional intelligence. Through the DMS Test for mean comparison, significant differences and similarities were determined among the groups. The assigned letters (A, B, C) denote the statistical significance between the means; means with the same letter show no significant differences at the 0.05 level.

**Table 4.** DMS analysis of mean comparison for age and mastery variables.

Factors and ranges	Attention	Clarity	Repair	IEP
Age (years)				
21 to 30	29.40 A1	29.87 C	31.04 C	90.33 A
31 to 40	27.92 B	30.55 BC	31.79 C	90.26 A
41 to 50	27.33 B	31.18 AB	32.20 AB	90.72 A
Over 50	28.32 AB	32.07 A	32.82 A	93.21 A
Mastery				
Education	26.79 B	30.26 B	31.65 A	88.71 B
Basic education	28.37 B	30.84 B	31.82 A	91.04 B
Early education	29.56 A	31.66 A	32.41 A	93.64 A

Note: Means with the same letter (A, B, C) in each column indicate that there are no statistically significant differences between them, according to the DMS Test criteria at a significance level of p<0.05.

A pivotal insight emanating from the conducted analysis pertains to the substantial correlation between emotional clarity and age. The existing corpus of research has consistently underscored the evolution of the capacity to interpret and comprehend emotions, a progression modulated by a confluence of biological underpinnings and accrued socio-emotional learning ( Averill, 1980; Mayer *et al*., 2016). The Chi-squared value of 22.27 manifested in this investigation fortifies this conceptualization, emphasizing that emotional clarity is a dynamic attribute, subject to vicissitudes across the chronological trajectory of an individual’s existence.

Concurrently, the discerned disparities in the dimensions of attention and clarity associated with sex, substantiated by Chi-squared values of 53.96 and 18.83, cohere with the existing scholarly discourse. This body of literature posits that variations exist in the ways men and women process and engage with emotions, potentially attributable to a matrix of sociocultural constructs and inherent biological determinants ( Averill, 1976; Meyers-Levy and Loken, 2015; Whittle et al., 2017). It is imperative to elucidate that such differences do not insinuate a hierarchical dichotomy or superiority of one over the other; rather, they signify diversification in the modalities of experiencing and conceptualizing emotions.

Furthermore, the discernible association between one’s field of mastery and the dimensions of emotional intelligence, corroborated by Chi-squared values of 13.34 and 9.93, intimates the influential role of academic training and experiential learning in sculpting emotional perception and discernment. It emerges as plausible that distinct academic disciplines, by virtue of their inherent characteristics and thematic emphasis, foster competencies germane to emotional recognition and clarity ( Chen and Lu, 2022; Denham and Brown, 2010).

**Figure 1.**
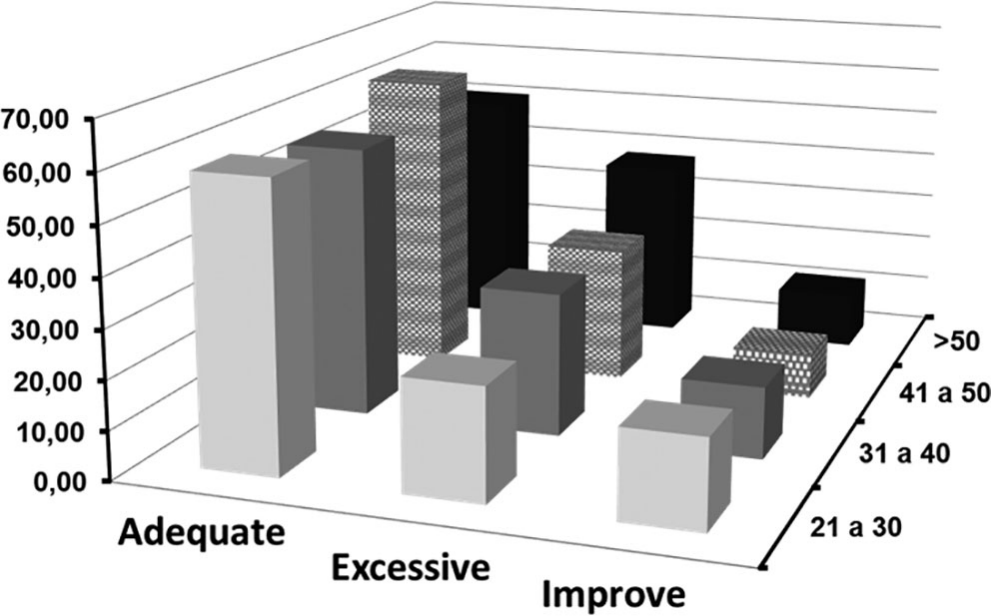
Frequencies of categorized clarity crossed with age.

Figure 1, presented below, illustrates this interaction. It provides a visual representation of the frequencies of categorized clarity according to different age cohorts. This type of graphical representation allows for a more intuitive interpretation of the data, highlighting trends, consistencies, or possible anomalies among the groups.

The graphical representation in Figure 1 highlights these temporal dynamics of emotional perception in relation to age. It is revealing to observe that, with advancing age, there is an increase in the “excessive” category. This trend could align with the idea that accumulated experiences and introspection throughout life enrich emotional perception ( Bukor, 2015). Older individuals may have a more finely-tuned ability to recognize and understand the depth and complexity of their emotions, resulting in an “excessive” perception of emotional clarity.

In contrast, the “improve” category exhibits a notable decline with age. This could be interpreted as increased confidence and certainty in emotional perception acquired over the years. The fact that this trend starts high in the youngest group and decreases with age suggests that, over time and experience, there is a consolidation of emotional understanding ( Casale *et al*., 2022).

However, while these trends provide a fascinating insight into the evolution of emotional clarity throughout life, it is essential, as in all research, to maintain a critical perspective. Individual differences, cultural factors, socioeconomic factors, and life experiences can modulate these patterns ( Gross and John, 1995; Seixas et al., 2021).

**Figure 2.**
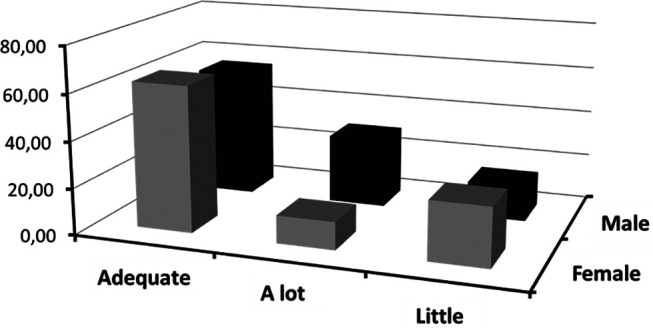
Frequencies of categorized attention crossed with sex.

Next, Figure 2 is presented, where a visual representation of the frequencies of categorized emotional attention based on sex is displayed. This graph provides a revealing perspective on how sex differences may be associated with variations in attention directed towards emotions.

In Figure 2, which illustrates the distribution of attention frequencies according to sex, distinctive patterns are observed in relation to the categories of emotional attention, mainly in the “adequate,” “little,” and “much” categories.

The female sex appears to predominate in the “adequate” and “little” categories, suggesting that females, in this study, tended to report levels of emotional attention that range between moderate and low. On the other hand, in the “much” category, a predominance of the male sex was observed, indicating that men in this sample report a high level of attention towards their own emotions or those of others.

These observations are consistent with literature in Psychology and Neuroscience that has explored sex differences in emotional perception and regulation. For example, Kashdan *et al*. (2009) posited that, in general, women may be more attuned to emotions, which could translate into more balanced or “adequate” levels of emotional attention. However, this does not necessarily imply that women pay less attention to emotions than men. Instead, it suggests that women may be more efficient in regulating the amount of attention they give to emotions, avoiding extremes.

On the other hand, the fact that men stand out in the “much” category could reflect a tendency among some men to be hyper-aware or excessively attentive to certain emotions. This pattern could be related to sociocultural norms that, in many contexts, discourage emotional expression among men, leading them to greater introspection and self-awareness ( Dworkin and Weaver, 2021).

However, it is crucial not to generalize these findings to all populations or interpret them as reflecting fixed biological differences between sex The observed differences may be influenced by sociocultural, educational factors, and individual life experiences.

**Figure 3.**
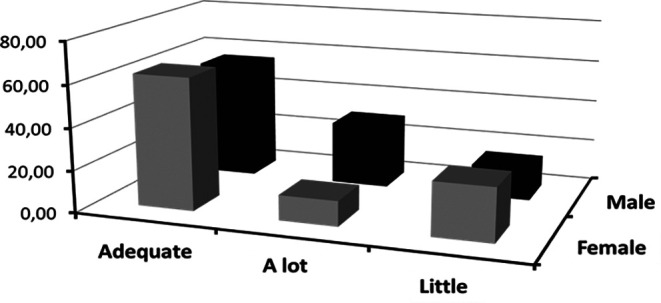
Frequencies of categorized emotional clarity crossed with sex.

In the following Figure 3, it is illustrated how the frequencies of categorized emotional clarity vary according to sex. This visualization will allow the exploration of whether there are distinctive patterns between sex concerning the clarity of their emotions.

In the field of emotional intelligence, emotional clarity denotes an individual’s ability to identify and understand their own emotions, an aspect that is of great importance as it provides insights into how people process and manage their emotions in daily life. Figure 3 offers a detailed view of the frequencies of categorized emotional clarity according to sex.

According to the data presented in Figure 3, it is observed that women tend to report higher frequencies of levels of emotional clarity considered “adequate” or “excessive” compared to men. However, in the “improve” category, men surpass women in frequency, suggesting a greater inclination on their part to feel that they need to improve in this aspect.

These differences can be interpreted from various perspectives. According to Doherty (1997), women tend to be more introspective and are socially conditioned to be more expressive and aware of their emotions, which could explain the higher frequencies in the “adequate” and “excessive” categories observed in females. In contrast, men, subject to certain sociocultural norms, might feel less encouraged to explore and understand their emotions, which could influence their perception of the need to “improve” in this area.

Additionally, it is interesting to highlight the hierarchy observed in the frequencies for both categories: “adequate”, “excessive”, and “improve”. These findings underline that, regardless of sex, most individuals feel that they possess an acceptable degree of emotional clarity. However, it is in the “improve” category where the most significant contrast between the sexes is found, prompting reflections on the differences in emotional self-assessment and the potential sociocultural influences underlying this perception.

Finally, it is imperative to emphasize that, while these findings are revealing, they should not be interpreted simplistically. Differences in the self-perception of emotional clarity can be multifactorial and, as suggested by Gomez-Baya *et al*. (2017), may be influenced by both biological and sociocultural factors. In this context, a detailed understanding of the observed patterns related to emotional clarity and sex opens doors for future research and reflections in the field of Emotional Psychology.

**Figure 4.**
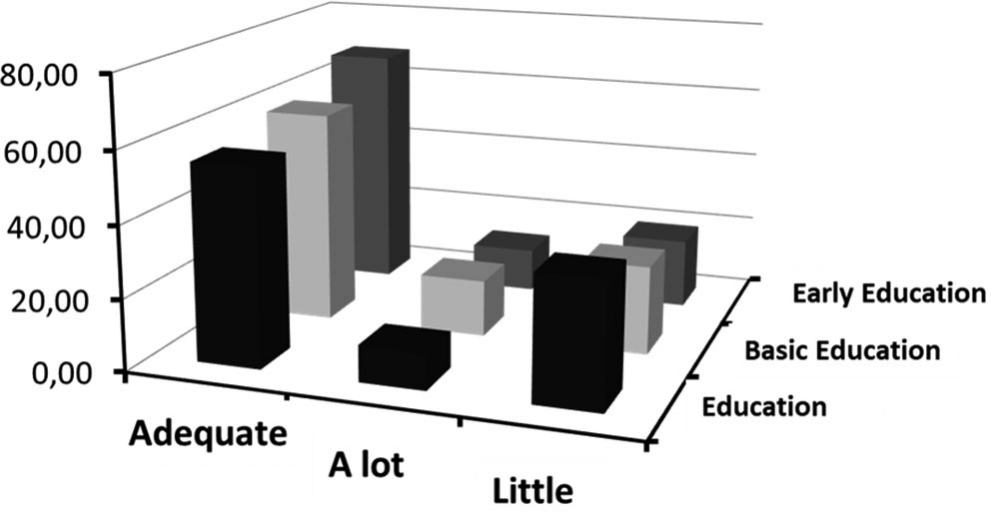
Frequencies of categorized clarity crossed with type of Master's program.

The following Figure 4 offers a revealing perspective on the interaction between emotional clarity and academic specialization at the Master’s level. Through this graphic representation, the aim is to analyze the possible influence of academic training on the perception and understanding of emotions among postgraduate students.

Emotional attention refers to an individual’s ability to tune into and be aware of their own emotions as well as those of others. This construct has crucial implications in the academic and professional world, as the way an individual pays attention to emotions can influence their learning and teaching abilities, as well as their capacity to interact with others in educational contexts.

According to Figure 4, a notable relationship is evident between attention categories and the type of Master’s program students are enrolled in. This relationship provides information on how different specializations may influence students’ self-perception of emotional attention.

A key finding observed is the gradient in the “adequate” category. Parada-Cabaleiro *et al*. (2020) argued that adequate perception of emotions is crucial for effective emotional processing. Students in “Early Childhood Education,” who often work with young children, might develop more finely-tuned emotional attention due to the intuitive and non-verbal nature of communication at early ages. This finding is corroborated by results obtained in studies like that of Joss *et al.* (2020), who argue that working with younger populations can cultivate greater sensitivity and attention to emotions.

On the other hand, the “low” category suggests a perception of insufficient emotional attention. Here, the highest frequency corresponds to students in the “Education” Master’s program. According to Jamil *et al.* (2023), traditional education often prioritizes cognitive skills over emotional skills. However, it is essential to consider that the type of population or educational context these programs target may influence these results.

Lastly, the “high” category presents an interesting pattern. The “Basic Education” Master’s holds the highest point, indicating that these students perceive elevated emotional attention. Aguado *et al.* (2018) argue that emotional attention can be influenced by the demands of the context. Basic education often requires a greater capacity to tune into a wide range of emotions due to the diversity of ages and issues.

**Figure 5.**
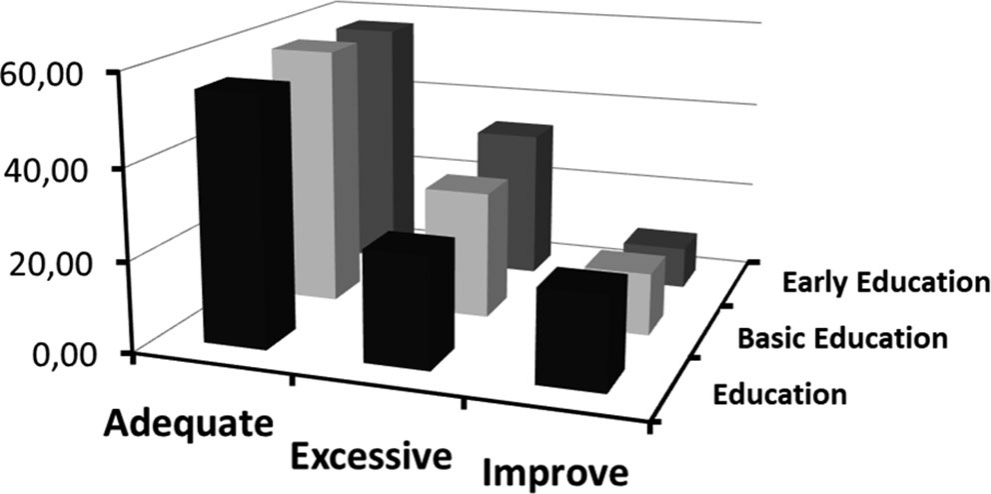
Frequencies of categorized clarity crossed with type of Master's program.

In Figure 5, the distribution of frequency categories of clarity, which have been crossed with different types of Master’s programs, is illustrated. This graphic representation aims to establish a relationship between the perception of clarity and different postgraduate students, allowing for a more detailed evaluation and a deeper interpretation of the collected data.

Figure 5 offers a detailed view of how emotional clarity relates to the academic specialization that students choose at the Master’s level. This graphical representation indicates a notable dependency between the perception of emotional clarity and the nature of the academic program being pursued.

Analysis of the graph reveals that the categories of “adequate” and “excessive” in emotional clarity exhibit a pattern of consecutive increase from students in “Education” to those in “Early Childhood Education” (“Ed. Inicial” in the original text). This finding is consistent with previous research that suggests that certain academic programs, especially those oriented towards initial training in education, may place a stronger emphasis on emotional development ( Hodson, 2003; McCoy and Wolf, 2018).

On the other hand, it is intriguing to note that the “improve” category reflects an inverse trend. Specifically, those students in the “Education” Master’s program show higher percentages, which could imply a greater awareness of their areas for improvement in emotional understanding or, alternatively, a program that has yet to fully emphasize this domain, as suggested by some authors ( Yang *et al*., 2022).

It is essential to point out that while these patterns may reflect the influence of curriculum and pedagogy on the perception of emotional clarity, they could also possibly reflect preexisting characteristics of the students who choose these programs. For example, those with intrinsically high emotional clarity may be more attracted to programs like “Early Childhood Education,” given their direct and profound interaction with child development ( Ryan and Deci, 2020).

## Conclusions

The present study embarked on the complex task of deciphering the relationship between demographic and emotional variables, a venture not without significant challenges. Through the use of rigorous quantitative methodologies, patterns of interaction between emotional clarity and attention in diverse populations were identified, shedding light on the complex dynamics underlying these emotional capacities.

A direct relationship between age and emotional clarity was evident; accumulated experiences and increased maturity significantly enhance individuals’ ability to interpret and manage their emotions. This finding reinforces the traditional view that associates wisdom with advancing age and suggests the possibility of designing interventions tailored to different age groups to optimize outcomes in terms of emotional development. Regarding gender differences, considerable disparities were observed in terms of emotional attention and clarity. However, the lack of robust statistical evidence to fully support these observations necessitates cautious interpretation of the results. It is plausible that these variations stem from a combination of sociocultural and biological factors, highlighting the importance of future research that delves deeper into these differences through more detailed statistical analysis and a comprehensive review of the related literature.

Furthermore, the study highlighted the significant impact of education at the master’s level in fostering introspection and emotional awareness. Advanced academic training not only improves emotional competencies but also strengthens the link between structured education and the development of emotional skills. Consequently, the implementation of specific pedagogical adjustments in academic settings is proposed to refine emotional clarity and attention, thus preparing future professionals to face emotional challenges with greater acuity and self-awareness.

The practical implications of these findings are far-reaching. It is essential for educational and therapeutic interventions to consider these demographic differences to avoid adopting generalized approaches that could prove ineffective. Professionals in the fields of education, therapy, and mental health must integrate these discoveries into their practices, which will allow the design of personalized strategies that enhance the effectiveness of the proposed interventions.

Although this study contributes significantly to academic discourse, its conclusions should not be considered definitive. Rather, the research acts as a catalyst for future inquiries, opening new lines of exploration on how additional socioeconomic or demographic factors might influence emotional clarity and attention. This analysis invites continued examination of the relationship between a diversity of influences and emotional intelligence, a critical task for understanding the complexities of human behavior in a globalized and constantly changing context.

## Data availability

Figshare: Data-Comparative Analysis of Psychological Well-being.xlsx. https://doi.org/10.6084/m9.figshare.24148155.v1 ( Moreira-Choez *et al*., 2023).

The project contains the following underlying data:

- Data-Comparative Analysis of Psychological Well-being.xlsx

Data are available under the terms of the Creative Commons Zero “No rights reserved” data waiver (CC BY 4.0 Public domain dedication).
