# Supplementary material for: Comparative Analysis of Psychological Well-Being and Emotional Education in Graduate Students
Source: F1000Res. 2024 Aug 6;12:1403. Originally published 2023 Oct 24. [Version 2] doi: 10.12688/f1000research.141849.2 (PMC11380077; doi:10.12688/f1000research.141849.2)
Supplement: Peer review history

# Reviewer report: Reviewer response for version 2

**DOI:** 10.5256/f1000research.166704.r311340

**Published:** 2024-09-12

**Authors:** Maira Alejandra Pulgarín Rodríguez

**Affiliations:** 1 Corporación Universitaria Minuto de Dios, Bogotá, Colombia

The new version of the paper contains the recommendations made by the reviewers.

Is the work clearly and accurately presented and does it cite the current literature?

Partly

If applicable, is the statistical analysis and its interpretation appropriate?

Yes

Are all the source data underlying the results available to ensure full reproducibility?

Yes

Is the study design appropriate and is the work technically sound?

Yes

Are the conclusions drawn adequately supported by the results?

Yes

Are sufficient details of methods and analysis provided to allow replication by others?

Yes

Reviewer Expertise:

Education process.

I confirm that I have read this submission and believe that I have an appropriate level of expertise to confirm that it is of an acceptable scientific standard.

# Reviewer report: Reviewer response for version 2

**DOI:** 10.5256/f1000research.166704.r318992

**Published:** 2024-09-06

**Authors:** Francis Britwum

**Affiliations:** 1 University of Cape Coast, Cape Coast, Ghana

Although the methodology is well-structured, there is  the need for more detail regarding the sample selection process. The author has to Provide a comprehensive descriptions of how the sample size was calculated, and the process involve in the sample selection which would enhance the replicability of the study.

 Some of the conclusions drawn in the study could benefit from stronger empirical backing. For example, while differences in emotional perception based on sex and academic fields are noted, the evidence supporting these findings should be more rigorously presented. Additionally, the implications for educational strategies could be elaborated upon, linking them directly to the findings.

 The document includes recent citations and references, but I suggest that the author incorporate more up-to-date studies (including those from 2023) to ensure the research reflects the latest developments and findings in the field.

 The study makes a significant contribution to understanding the role of emotional education in the psychological well-being of graduate students. It is methodologically sound, and its findings are relevant for educational and psychological interventions. However, improvements can be made in the depth of methodological transparency, and the strength of the conclusions drawn. Addressing these areas will enhance the study's rigor and impact. Thank you

Is the work clearly and accurately presented and does it cite the current literature?

Yes

If applicable, is the statistical analysis and its interpretation appropriate?

Yes

Are all the source data underlying the results available to ensure full reproducibility?

Yes

Is the study design appropriate and is the work technically sound?

Yes

Are the conclusions drawn adequately supported by the results?

Partly

Are sufficient details of methods and analysis provided to allow replication by others?

Partly

Reviewer Expertise:

educational Psychology

I confirm that I have read this submission and believe that I have an appropriate level of expertise to confirm that it is of an acceptable scientific standard, however I have significant reservations, as outlined above.

# Reviewer report: Reviewer response for version 2

**DOI:** 10.5256/f1000research.166704.r282514

**Published:** 2024-08-14

**Authors:** Adriana Beatriz Sánchez-Urdaneta

**Affiliations:** 1 Universidad Tecnica de Manabi, Portoviejo, Manabí Province, Ecuador

Through this message I indicate the final approval of the article - Cooperative analysis of psychological well-being and emotional education in graduate students of Moreira-Choez Jenniffer Sobeida, Lemus de Rodríguez Tibisay Milene, Espinoza-Solís Eduardo Javier and Castro-Castillo Graciela Josefina.

 After reviewing the comments made to the manuscript, it can be seen that the authors accepted the observations made, therefore, as far as I am concerned, I approve of the changes made and that they contributed to improve the quality of the manuscript.

Is the work clearly and accurately presented and does it cite the current literature?

Partly

If applicable, is the statistical analysis and its interpretation appropriate?

Yes

Are all the source data underlying the results available to ensure full reproducibility?

Yes

Is the study design appropriate and is the work technically sound?

Partly

Are the conclusions drawn adequately supported by the results?

Partly

Are sufficient details of methods and analysis provided to allow replication by others?

Partly

Reviewer Expertise:

NA

I confirm that I have read this submission and believe that I have an appropriate level of expertise to confirm that it is of an acceptable scientific standard.

# Reviewer report: Reviewer response for version 1

**DOI:** 10.5256/f1000research.155333.r218145

**Published:** 2024-05-16

**Authors:** Maira Alejandra Pulgarín Rodríguez

**Affiliations:** 1 Corporación Universitaria Minuto de Dios, Bogotá, Colombia

The study conducted by Moreira et al. constitutes an important contribution to the study of sustainability in the context and well-being of students in higher education. The objective of the research, which was to compare the perceived emotional intelligence in different programs, can be clearly identified. Thus, enlightening results were obtained through the application of the quantitative method that sought to examine the responses of 1,522 university students using the Trait Meta-Mood Scale (TMMS-24).

 Although carrying out quantitative studies allows to recognize a factual vision of the object of study, it is necessary to make an exhaustive tracking of the same that allows to have a global context of what is analyzed, in that case in the introduction it is required to make an extension in the existing foundations regarding the categories defined for the study, only three referents are located, although it is a novel topic there are different optics that could give a better support for what they present later in the method and the results. Likewise, the conclusions of the study do not have any reference that allows validating or corroborating a previously established theory or if new knowledge would be generated.

Is the work clearly and accurately presented and does it cite the current literature?

Partly

If applicable, is the statistical analysis and its interpretation appropriate?

Yes

Are all the source data underlying the results available to ensure full reproducibility?

Yes

Is the study design appropriate and is the work technically sound?

Yes

Are the conclusions drawn adequately supported by the results?

Yes

Are sufficient details of methods and analysis provided to allow replication by others?

Yes

Reviewer Expertise:

Education process.

I confirm that I have read this submission and believe that I have an appropriate level of expertise to confirm that it is of an acceptable scientific standard, however I have significant reservations, as outlined above.

# Reviewer report: Reviewer response for version 1

**DOI:** 10.5256/f1000research.155333.r218147

**Published:** 2023-11-21

**Authors:** Adriana Beatriz Sánchez-Urdaneta

**Affiliations:** 1 Universidad Tecnica de Manabi, Portoviejo, Manabí Province, Ecuador

I consider that the bibliography used partially corresponds to the research. Although relevant references on emotional intelligence and psychological well-being are included, there is a need to go deeper into specific studies on emotional education in graduate students, which is what the study refers to. On the other hand, I also consider that some paragraphs need to support the information provided with certain bibliographical references. It is also important to point out that, although a high percentage of the bibliography used is updated in the last 5 years, only one reference corresponding to 2023 is used.

 It is true that this is a quantitative study with a considerably large sample and validated instruments are used, which is appropriate. However, I believe it is necessary to expand on the details of the sample selection process and the statistical analyses performed. I consider that the methods and analyses are not described in sufficient detail to allow an exact replication of the study.

 It is also important to note that the authors have followed appropriate guidelines regarding informed consent of the participants and approval by an ethics committee. However, I believe it is unclear whether specific guidelines were followed in the development and reporting of the quantitative research.

 As for statistical analysis, appropriate tests such as ANOVA, Pearson correlations and descriptive statistics are used. However, more detail is needed on the specific procedures and parameters used in these tests in order to adequately evaluate their implementation.

 The source data according to the information provided are available, so with the results presented in tables and figures and the data, the reproducibility of the information is feasible.

 Regarding the conclusions presented, they are partially based on the results, making very general statements about the differences between sex and age that would require a more robust support from the statistical analysis. For example, differences between men and women are mentioned, but no statistical evidence is reported to validate these differences. Nor do they support them through the use of bibliographic references related to this aspect.

 The discussion acknowledges some limitations regarding the possible influence of cultural factors and individual experiences. However, important limitations such as the cross-sectional design of the study are not discussed.

 In summary, the study has a solid technical basis, but I consider that it is necessary to deepen the bibliography, to better detail the methods and to strengthen the information generated by the study. On the other hand, the statistical analysis seems appropriate, but lacks detail. The conclusions are not always solidly supported by the results presented. Further discussion of methodological limitations is required. I believe that, with these adjustments, the article could provide interesting results, and the solidity and reproducibility of the article on a relevant topic such as emotional education in graduate students would be improved.

Is the work clearly and accurately presented and does it cite the current literature?

Partly

If applicable, is the statistical analysis and its interpretation appropriate?

Yes

Are all the source data underlying the results available to ensure full reproducibility?

Yes

Is the study design appropriate and is the work technically sound?

Partly

Are the conclusions drawn adequately supported by the results?

Partly

Are sufficient details of methods and analysis provided to allow replication by others?

Partly

Reviewer Expertise:

NA

I confirm that I have read this submission and believe that I have an appropriate level of expertise to confirm that it is of an acceptable scientific standard, however I have significant reservations, as outlined above.

# Response

**Published:** 2024-05-17

**Authors:** Jenniffer Sobeida Moreira Choez

Distinguished reviewer,

 Thank you for your valuable suggestions that have significantly contributed to improving the quality of our article. Below, we describe the changes made in response to your recommendations:

1. **In-depth focus on specific studies about emotional education in graduate students**: We have enriched the theoretical framework by incorporating current research that directly addresses emotional education in graduate contexts. Recent studies have been added to provide a more solid and contextualized foundation on the relevance of the topic in the current academic landscape.
2. **Support with additional bibliographic references**: We have enhanced the bibliographic support in key sections of the manuscript by integrating recent and relevant academic sources to strengthen the claims and conclusions derived from our data analysis. This approach ensures that all significant statements are backed by relevant and up-to-date empirical evidence.
3. **Details on the sample selection process and statistical analyses**: We provided detailed descriptions of the sample design and analytical procedures, including details on the sample size calculations and specific statistical techniques used, such as ANOVA and Pearson correlations. This level of detail is essential to allow for the replication of the study and to enhance methodological transparency.
4. **Clarity in ethical guidelines and informed consent**: Information on compliance with ethical standards has been expanded, detailing procedures for informed consent and ethical approval of the study, which is crucial to ensure the integrity and ethics of the research process.
5. **Robustness of statistical analysis and caution in conclusions**: The study's conclusions were revised to reflect a more conservative interpretation based on the data of observed differences by sex and age, highlighting the need for further research to allow a more robust validation of these findings.
6. **Discussion on the limitations of the cross-sectional design**: A critical discussion on the limitations imposed by the cross-sectional design used has been integrated, emphasizing the importance of future longitudinal studies to confirm and extend the presented findings.

These adjustments have been made to ensure that the study not only responds to the specific needs identified by your review but also enhances its contribution to the field of emotional education in graduate contexts. We appreciate your essential input in the review process and hope that the changes made meet the expectations of rigor and clarity required for the publication and indexing of our work.
